# Rotavirus A infection in children under five years old with a double health problem: undernutrition and diarrhoea – a cross-sectional study in four provinces of Mozambique

**DOI:** 10.1186/s12879-020-05718-9

**Published:** 2021-01-06

**Authors:** Assucênio Chissaque, Marta Cassocera, Carolina Gasparinho, Jéronimo Souzinho Langa, Adilson Fernando Loforte Bauhofer, Jorfélia José Chilaúle, Eva Dora João, Benilde António Munlela, Júlia Assiat Monteiro Sambo, Simone Salvador Boene, Marlene Bernardo Djedje, Elda Muianga Anapakala, Esperança Lourenço Guimarães, Diocreciano Matias Bero, Lena Vânia Manhique-Coutinho, Idalécia Cossa-Moiane, Timothy A. Kellogg, Luzia Augusta Pires Gonçalves, Nilsa de Deus

**Affiliations:** 1 Instituto Nacional de Saúde (INS), Maputo, Moçambique; 2 Instituto de Higiene e Medicina Tropical (IHMT), Universidade Nova de Lisboa, Lisboa, Portugal; 3 Global Health and Tropical Medicine, Instituto de Higiene e Medicina Tropical (IHMT), Universidade Nova de Lisboa, Lisboa, Portugal; 4 Centro de Biotecnologia - Universidade Eduardo Mondlane, Maputo, Moçambique; 5 Departamento de Ciências Biológicas, Universidade Eduardo Mondlane, Maputo, Moçambique; 6 Institute of Tropical Medicine (ITM), Antwerp, Belgium Institute for Global Health Sciences, Antwerp, Belgium; 7 University of California San Francisco, San Francisco, California USA; 8 Unidade de Saúde Pública Internacional e Bioestatística, Instituto de Higiene e Medicina Tropical, Universidade Nova de Lisboa, Lisboa, Portugal; 9 Centro de Estatística e Aplicações da Universidade de Lisboa, Lisboa, Portugal

**Keywords:** Undernutrition, Rotavirus A, Diarrhoea, Risk factors, Mozambique

## Abstract

**Background:**

Mozambique has a high burden of group A rotavirus (RVA) infection and chronic undernutrition. This study aimed to determine the frequency and potential risk factors for RVA infection in undernourished children under 5 years old with diarrhoea in Mozambique.

**Methods:**

The analysis was conducted using data from March 2015 to December 2017, regarding children under 5 years old with at least one type of undernutrition. Anthropometric measures were used to calculate indices of weight-for-age, weight-for-height and height-for-age through the Z-Scores. RVA results were extracted from the National Diarrhoea Surveillance database. Descriptive statistics, chi-square test was used for qualitative variables and organized in contingency tables and 95% Confidence Intervals (CI) were considered for the calculation of RVA infection proportion and in the multiple logistic regression models to estimate the adjusted odds ratios (AOR).

**Results:**

Of the 842 undernourished children included in the analysis, 27.2% (95% CI: 24.3–30.3%) were positive for RVA. The rate of RVA infection was 42.7% (95% CI: 38.0–47.5%) in the pre-vaccine period, with great reduction to 12.2% (95% CI: 9.4–15.6%) in the post-vaccine period. Most of the RVA undernourished children had severe wasting (33.3%) and severe stunting (32.0%). The risk of infection was significantly high in children from 0 to 11 months (*p*-value < 0.001) when compared to the age group of 24–59 months. A higher proportion of RVA infection was detected in households with five or more members (*p*-value = 0.029). Similar proportions of RVA were observed in children fed only by breast milk (34.9%) and breast milk with formula (35.6%). A higher proportion of undernourished HIV-positive children co-infected with RVA (7.4%) was observed.

**Conclusions:**

The frequency of RVA infection in undernourished children declined following the introduction of the vaccine in Mozambique. Beyond the temporal variation, Maputo province, age and crowded households were also associated to RVA infection. A high proportion of RVA infection was observed in children with severe wasting and a triple burden of disease: undernutrition, RVA and HIV, highlighting the need to conduct follow-up studies to understand the long-term impact of these conditions on children’s development.

## Background

Group A rotavirus (RVA) infection is a major cause of diarrhoea in children under 5 years old worldwide [1, 2]. Between 2000 and 2013, RVA-associated mortality declined from 528,000 to 215,000 deaths as a result of the introduction of RVA vaccine in the National Immunization Programs of several countries, as recommended by the World Health Organization (WHO) since 2006 [3]. Despite the efforts made over the years, RVA remains one of the leading causes of morbidity and mortality, and is responsible for approximately 104,733 deaths in children under 5 years old living in sub-Saharan Africa, where Mozambique is located [4].

Undernutrition is also a significant health problem in low and middle-income countries such as Mozambique, where RVA has a high burden [5]. Data from 2018 showed that more than 50% of all stunted children worldwide were living in Asia and Africa, and the latter continent contributes 39% [5]. Undernutrition can lead to impaired growth and poor development of children. This condition can result from the inadequate dietary intake, contributing to weight loss, an impaired immune response to pathogens, mucosal damage, intestinal inflammation, and lower absorption capacity as a consequence of invasion by enteric pathogens [6].

Studies conducted in Angola and Bangladesh observed an association between different types of undernutrition with RVA infection [7, 8]. However, this association remains a controversial topic given the divergent results found in the literature. A birth cohort study of 626 infants in Bangladesh demonstrated an association between RVA infection and well-nourished children during the first 3 years of life [9]. In another study in Zambia, the proportion of RVA-positive children was higher in well-nourished than in undernourished children (27.6% versus 19.3%) [10]. Some authors have hypothesized that undernutrition can reduce the efficacy of RVA vaccine, especially in countries with high levels of undernutrition [11]. In Ghana, a two-year randomized controlled trial study found that RVA vaccine efficacy was lower in underweight children than in those well-nourished [11].

Mozambique has a high burden of undernutrition in children under five [12]. The Demographic and Health Survey (DHS) conducted in 2011 estimated 43% of stunting prevalence among children under 5 years [12]. The most affected areas of the country were the northern provinces of Nampula and Cabo-Delgado, with 55% and 52%, respectively. Although stunting was reported to be higher in rural areas (46%), it was also high in urban environments (35%) [12].

Studies conducted in Manhiça district, southern Mozambique, showed an association between diarrhoea and undernutrition [13, 14]. During 10 years of surveillance, 47% of the hospitalized children presented one or more types of undernutrition, of which 6% were severely undernourished [13]. Also in the same area, another study reported undernutrition as one of the risk factors for mortality in children with moderate-severe-diarrhoea [14]. To date, most studies of diarrhoeal diseases took place in southern Mozambique, with few studies conducted exclusively in northern provinces [15].

Mozambique introduced the G1P [8] RVA vaccine (Rotarix®; GlaxoSmithKline Biologics, Belgium) to the national immunization program in September 2015. This vaccine is administrated to children in two doses: the first at the second month of age and the second dose in the third month [16]. Before the vaccine introduction, the frequency of RVA infection in children ranged from 24% to 40.2% in different studies [17–19]. Following RVA vaccine introduction, the frequency reduced to 12.2% in 2016 and 13.5% in 2017. However, there are RVA cases still reported in the country [20].

In 2014, the *Instituto Nacional de Saúde* (INS) - Mozambique implemented a hospital-based surveillance system (National Diarrhoea Surveillance - ViNaDia) to track diarrhoeal illness among children. This system provides an opportunity to measure and describe nutritional status and enteric agents among children with diarrhoea. In this study, we aim to determine the frequency of RVA infection and potential risk factors in undernourished children in four provinces of Mozambique before and after the introduction of the RVA vaccine in the country.

## Methods

### Design and setting of the study

A cross-sectional analysis was conducted using data from ViNaDia surveillance system to determine the frequency and risk factors of RVA infection in undernourished children under 5 years. Which were admitted with diarrhoea in the ViNaDia sentinel sites during the pre-vaccine (March–December 2015) and post-vaccine periods (January 2016–December 2017). ViNaDia has six hospitals serving as sentinel sites located within four provinces of Mozambique. These locations are: *Hospital Central de Maputo* (HCM), *Hospital Geral de Mavalane* (HGM) and *Hospital Geral José Macamo* (HGJM), all located in Maputo province. *Hospital Central da Beira* (HCB) in Sofala; *Hospital Geral de Quelimane* (HGQ) in Zambézia, and *Hospital Central de Nampula* (HCN) in Nampula. ViNaDia’s methodology has been previously described [20, 21].

### Study population

ViNaDia included children admitted for diarrhoea as a primary symptom, defined as three or more episodes of diarrhoea in less than 24 h [22]. For the present analysis, children were considered eligible according to the following criteria: (i) age under 5 years; (ii) children suffering from at least one type of undernutrition, mild-to-severe stunting, mild-to-severe wasting, or mild-to-severe underweight; (iii) consent signed by caregivers, allowing the children to participate in the study; (iv) children with RVA diagnosis available in the database. Children were excluded from this analysis if age, gender, weight or height were missing because these variables are required to measure nutritional status. Trained health professionals collected the stool samples, socio-demographic data and clinical information including children HIV status from patient records, vaccination cards and caretakers interviews using a structured data collection form.

### Sample size

The sample size was calculated using Epitools [23]*.* To our knowledge, this is the first study in Mozambique reporting RVA infection and related risk factors in undernourished children. For this reason, we considered the expected prevalence of 50%, which gives a maximum sample. Considering a 95% CI and assuming an acceptable error of 0.036, a minimum of 742 children is required. We increased the sample size assuming a 10% of non-response compensation rate. Thus, at least 816 undernourished children with diarrhoea should be included in the present analysis.

### Nutritional assessment

Trained health professionals of the ViNaDia assessed anthropometric measurements and recorded data on standardized data collection forms. The length for children under 2 years of age was measured in a recumbent position lying down, while children two or more years of age, the height was measured while standing [24].

Nutritional status was determined using the World Health Organization (WHO) Anthro V3.2.2 program [25]. The child’s nutritional status was obtained by calculating: Weight-for-Age Z-scores (WAZ) to determine underweight status, Weight-for-height Z-scores (WHZ) to determine wasting status, and Height-for-age Z-scores (HAZ) to determine stunting status. The classification of the child’s underweight, wasting, and stunting was made using the WHO tables [25].

The degree of undernutrition was defined using the following parameters: (i) adequate scores (− 1 ≤ Z-score ≤ + 1); (ii) mild undernutrition (− 2 ≤ Z-scores < − 1); (iii) moderate undernutrition (− 3 ≤ Z-score < − 2), and (iii) severe undernutrition Z-score < − 3 [25]. Children whose nutritional status was classified as adequate or overweight (Z-score > 1) were excluded from the analysis [25]. Some flags that are implausible values that are out of the default values of z-scores were considered, for underweight and stunting the flags were < − 6 and for wasting < − 5.

### Sample collection

Through the ViNaDia surveillance system, each child provided a single stool sample. The stool sample were collected into a 50 ml sterile vial, which was transported to the *INS* central laboratory located in Maputo for testing. For the sites located in Maputo (HGM, HCM, HJM), cooler boxes with ice packs were used for sample transportation, which was done by car and took approximately 2 h of traveling. For the sites outside of Maputo (HGQ, HCB, and HCN), samples were collected and stored at − 20 °C until shipment by plane in a cooler boxes with ice packs to the INS. After arrival, all samples were stored at − 40 °C in the central laboratory at INS in Maputo until testing for RVA.

### Laboratory diagnosis

Stool samples were tested for RVA identification by Enzyme Linked ImmunonoSorbent Assay (ELISA) using a commercial kit (ProSpecT™ Rotavirus, Oxoid, United Kingdom) according to the manufacturer’s recommendations [26]. The cut-off value was calculated by adding 0.200 absorbance units to the negative control value.

### Data management

Demographic, clinical, and laboratory data were double-entered using the Epi Info™ V3.5.1 program (Centers for Disease Control and Prevention, Atlanta, 2008) [27] and possible inconsistencies were identified and corrected generating a final database for analysis.

### Statistical analysis

Data analysis were performed using Statistical Package for Social Sciences (SPSS), Armok, NY: IBM Corp., 2011, version 25.0. The presence or absence of RVA infection was considered the outcome of interest.

Descriptive statistics for demographic and clinical characteristics were performed. Cross tabulations of characteristics and predictor variable with the dependent variable were constructed. Contingency tables were evaluated by chi-square test or Fisher’s exact test for qualitative variables. Confidence intervals (95% CI) by the Wilson method, were calculated to proportions of RVA for global, pre-vaccine, and post-vaccine periods, using EpiTools [23, 28]. After an initial descriptive analysis, simple and multiple logistic regression models were explored. Multiple models included variables whose *p*-values ​​were below 0.20 in simple logistic models. Thus, we obtained the crude odds ratio (OR), using simple models, and Adjusted Odds Ratio (AOR) with 95% CI using multiple logistic regression models, controlling for possible confounding factors. The Hosmer-Lemeshow was used to test the goodness of fit for the multiple logistic regression models at a significance level of 0.05.

## Results

During the study period (March 2015 – December 2017), 1331 children under 5 years of age with diarrhoea were enrolled in the ViNaDia surveillance system, of which 82.6% (1099/1331) provided a single stool sample for testing. Of these, 842 (76.6%) were assessed with at least one type of undernutrition and thus were included in the present analysis. The remaining 257 children were excluded for several reasons: 50 had insufficient/inadequate stool sample and 207 were well-nourished; those that undernutrition diagnosis was not possible, and those whose Z-scores flags were not eligible for the analysis.

### Socio-demographic characteristics

As presented in Table 1, 51.4% of the children were under 12 months of age and 56.2% were male. The highest percentage of children were recruited in 2015 (49.3%) during the pre-vaccine period. The majority number of children were admitted in Maputo (50.8%) followed by Nampula province (38.5%). Additionally, 58.6% of households had five or more family members in the household and 57.1% of the children lived in brick houses. Regarding the type of food, the frequency of exclusive breastfeeding in children under 6 months of age was 45.5%, while a combination of breast milk and formula was performed by 36.5% of children between 6 and 24 months of age.

**Table 1:** Socio-demographic characteristics of undernourished children

Variable	Categories	***N*** = 842	%
**Age in months**	0–11	433	51.4
	12–23	300	35.6
	24–59	109	12.9
**Sex**	Male	473	56.2
	Female	369	43.8
**Vaccine period**	Pre-vaccine (2015)	415	49.3
	Post-vaccine (2016–2017)	427	50.7
**Year of admission**	2015	415	49.3
	2016	317	37.6
	2017	110	13.1
**Province**	Maputo	428	50.8
	Sofala	42	5.0
	Nampula	324	38.5
	Zambézia	48	5.7
**Number of family members in the household**	<5	306	36.3
	≥5	493	58.6
	Unknown/ missing	43	5.1
**Number of children under five in the household**	<5	723	85.9
	≥5	77	9.1
	Unknown/ missing	42	5.0
**Type of house**	Brick	481	57.1
	Mud and Reed	289	34.3
	Unknown/ missing	72	8.6
**Care giver age**	≥20	139	16.5
	21–30	449	53.3
	> 30	220	26.1
	Unknown/ missing	34	4.0
**Type of food by age groups**	**Exclusive breastfeeding**	**218**	**25.9**
	0–5 months	35	45.5
	6–24 months	182	27.8
	≥25 months	1	1.1
	**Breastfeeding and formula**	**292**	**34.7**
	0–5 months	13	16.9
	6–24 months	239	36.5
	≥25 months	40	42.1
	**Formula only**	**41**	**4.9**
	0–5 months	16	20.8
	6–24 months	22	3.4
	≥25 months	3	3.2
	**Other**	**275**	**32.7**
	0–5 months	13	16.9
	6–24 months	211	32.3
	≥25 months	51	53.7
	Unknown/ missing	16	1.9

### Nutritional status and clinical condition

As shown in Table 2, 18.1% of children were suffering from wasting, 84.1% were underweight while 18.7% were stunted. About 40% of children experienced four to five episodes of diarrhoea, while 59.3% had also vomiting, almost 7% of them were infected with the Human Immunodeficiency Virus (HIV). More than one-third (34.3%) were hospitalized from 1 to 4 days (Table 2).

**Table 2:** Nutritional status and clinical profile of children included

Characteristic	Categories	***N*** = 842	%
**Types of undernutrition**			
**Wasting**	Mild-to-moderate	110	13.1
	Severe	42	5.0
**Underweight**	Mild-to-moderate	708	84.1
	Severe	0	0.0
**Stunting**	Mild-to-moderate	108	12.8
	Severe	50	5.9
**Diarrhoea episodes in 24 h**	1–3	238	28.3
	4–5	332	39.4
	≥6	196	23.3
	Unknown/ missing	76	9.0
**Vomit**	Yes	499	59.3
	No	337	40.0
	Unknown/ missing	6	0.7
**HIV status**	Positive	55	6.5
	Negative	518	61.5
	Unknown/ missing	269	31.9
**Duration of hospitalization (days)**	1–4	289	34.3
	5	77	9.1
	≥6	160	19.0
	Unknown/ missing	316	37.5

**Fig. 1:**
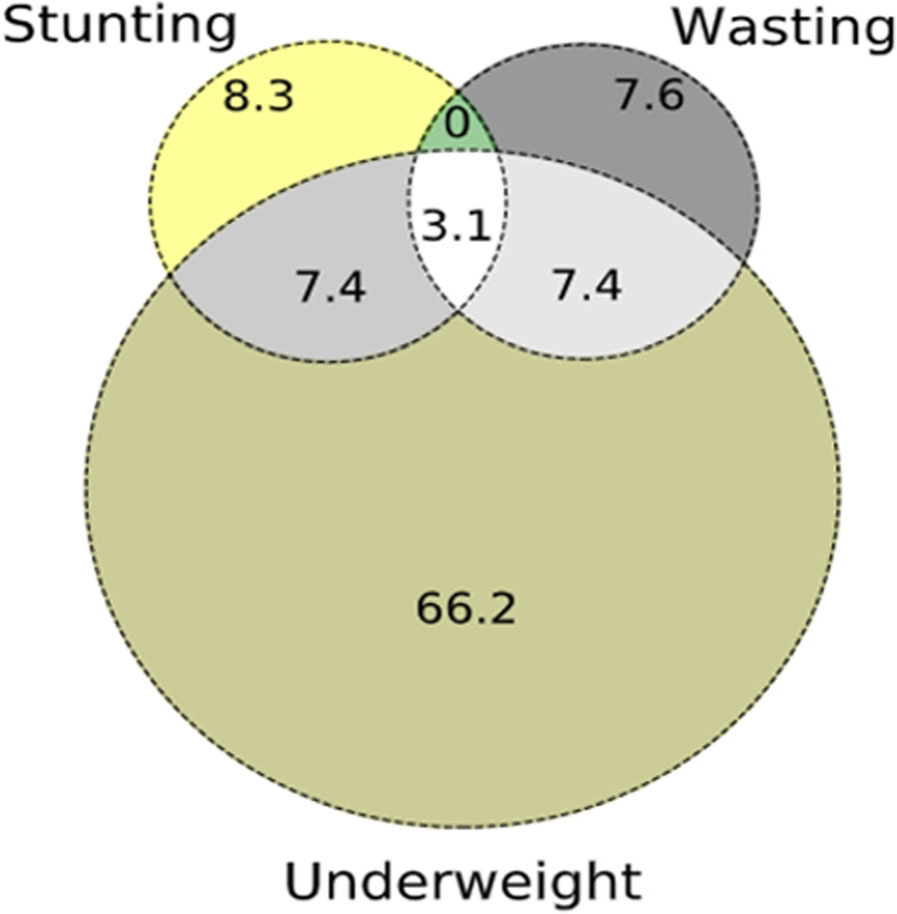
The Venn diagram shows the relationship between different nutritional indicators in undernourished children

We observed a large percentage of children with underweight (66.2%); 8.3% with stunting and 7.6% suffering from wasting. An overlap between different nutritional indicators was observed: 7.4% between underweight and stunting; 7.4% between underweight and wasting and 3.1% between underweight, stunting and wasting (Fig. 1).

### Frequency of RVA infection in undernourished children in pre- and post-vaccine era

**Fig. 2:**
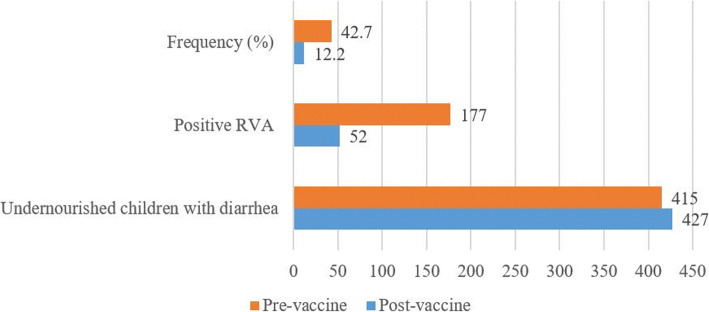
Frequency of RVA infection in pre- and post-vaccine period among undernourished children

During the study period, 27.2% (95% CI: 24.3–30.3%; 229/842) of the children were positive for RVA. In 2015, during the pre-vaccine era, the RVA rate was 42.7% (95% CI: 38.0–47.5%; 177/415) (Fig. 2). Two years after RVA vaccine introduction, the rate of positive reduced to 12.2% (95% CI: (9.4–15.6%; 52/427). By year, 13.2% (95% CI: 10.0–17.4%; 42/317) - in 2016 and 9.1% (95% CI: 5.0–15.9%; 10/110) - in 2017 (Fig. 2).

According to the fitted model, undernourished children in pre-vaccine period (2015) were 16 times more likely to be infected with RVA than in the second year of post-vaccine (2017) (AOR = 16.60; 95% CI: 6.54–42.14; *p*-value < 0.001).

### Socio-demographic characteristics of RVA-positive undernourished children and risk factors

The frequency of RVA infection was higher in children less than 12 months of age (31.6%), followed by the group of children between 12 and 23 months (27%) and those between 24 and 59 months (10.1%) (Table 3).

**Table 3:** Demographic characteristics, frequencies, crude and adjusted odds ratio for undernourished children infected by RVA

Demographic characteristics	***N*** = 842	Positive RVA (***n*** = 229)	% (Pos)	***P***-value	Crude OR (95% CI)	***P***-value	Adjusted OR (95% CI)
**Age in months**				< 0.001		< 0.001	
0–11	433	137	31.6	< 0.001	4.12 (2.14–7.94)	< 0.001	4.39 (2.08–9.25)
12–23	300	81	27.0	0.001	3.30 (1.68–6.46)	0.005	2.93 (1.38–6.23)
24–59	109	11	10.1		**Ref**		**Ref**
**Sex**							
Male	473	137	29.0	0.192	1.23 (0.90–1.67)	0.376	1.18 (0.82–1.70)
Female	369	92	24.9		**Ref**		**Ref**
**Year of admission**				< 0.001		< 0.001	
2015	415	177	42.7	< 0.001	7.44 (3.77–14.66)	< 0.001	16.60 (6.54–42.14)
2016	317	42	13.2	0.253	1.53 (0.74–3.16)	0.040	2.23 (1.04–4.77)
2017	110	10	9.1		**Ref**		**Ref**
**Province**				0.003		0.115	
Maputo	428	137	32.0	0.010	3.48 (1.34–9.06)	0.019	3.38 (1.22–9.37)
Nampula	324	80	24.7	0.073	2.43 (0.92–6.38)	0.071	2.65 (0.92–7.63)
Zambézia	48	7	14.6	0.710	1.26 (0.37–4.33)	0.288	2.10 (0.53–8.27)
Sofala	42	5	11.9		**Ref**		**Ref**
**Family members in household**							
<5	306	62	20.3		**Ref**		**Ref**
≥5	493	160	32.5	< 0.001	1.89 (1.35–2.65)	0.029	1.55 (1.05–2.29)
Unknown/ missing	43	7	–				
**Number of children under five**							
< 5	723	194	26.8		**Ref**	**–**	**–**
≥ 5	77	30	39.0	0.024	1.74 (1.07–2.83)	**–**	**–**
Unknown/ missing	42	5	–				
**Type of house**				0.001		0.497	
Brick	481	161	33.5		1.73 (1.24–2.42)		1.20 (0.71–2.03)
Mud and Reed	289	65	22.5		**Ref**		**Ref**
Unknown/ missing	72	3	–				
**Care giver age**				0.841			
≥ 20	139	39	28.1	0.980	0.99 (0.62–1.59)	**–**	**–**
21–30	449	118	26.3	0.602	0.91 (0.63–1.30)	**–**	**–**
> 30	220	62	28.2		**Ref**		
Unknown/ missing	34	10	–				
**Type of food**				< 0.001		0.173	
Breast milk	218	76	34.9		**Ref**		**Ref**
Breast milk and formula	292	104	35.6	0.860	0.97 (2.27–5.24)	0.411	1.21 (0.77–1.91)
Formula	41	9	22.0	0.088	0.51 (0.78–3.96)	0.732	0.85 (0.34–2.15)
Other	275	38	13.8	< 0.001	0.29 (0.19–0.44)	0.042	2.22 (1.03–4.80)
Unknown/ missing	16	2	–				

*Ref* Reference category

-: Not applicable

Hosmer and Lemeshow test, *p*-value = 0.318

Children under 12 months of age have shown to be four times more likely to be infected by RVA than children between 24 and 59 months of age (AOR = 4.39; 95% CI: 2.08–9.25; *p*-value < 0.001). Older children aged 12 to 23 months had almost three times more chances to be infected by RVA if compared to the oldest age group (AOR = 2.93; 95% CI: 1.38–6.23; *p*-value = 0.005) according to Table 3.

The frequency of infection was higher in male children (29.0% - 137/473) than in females (24.9% - 92/369), although the difference was not statistically significant (AOR = 1.18; 95% CI: 0.82–1.70; *p*-value = 0.376) (Table 3).

The frequency of RVA varied by province; Maputo had a higher frequency of infection (32% - 137/428), followed by Nampula (24.7% - 80/324), Zambézia (14.6% - 7/48) and Sofala (11.9% - 5/42). Additionally, undernourished children living in Maputo were three times more likely to be infected by RVA when compared to those living in Sofala province (AOR = 3.38; 95% CI: 1.22–9.37; *p*-value = 0.019) (Table 3).

Children living in households with five or more family members had a higher frequency of infection (32.5% - 160/493) than those from households with fewer members (20.3% -62/306) (AOR =1.55; 95% CI: 1.05–2.29; *p*-value = 0.029) (Table 3).

The frequency of RVA infection was higher in households with at least five children less than 5 years old (39% - 30/77) compared with those households with fewer children (26.8% - 194/723). Although the number of children in the household was significant in bivariate analysis, this variable was not included in the final model due to its possible correlation with the number of household family member’s variable.

Children living in brick houses had a higher frequency of RVA infection 33.5% (161/481) than those living in mud or reed houses (22.5% - 65/289) although this difference was not statistically significant (Table 3).

Regarding feeding, it was found a similar frequency of RVA infection in children fed by a combination of breast milk and formula (35.6% - 104/292) and by exclusive breastfeeding 34.9% (76/218) (Table 3). The adjusted regression model suggested that children fed with other types of food have a high risk of RVA infection (AOR = 2.22; 95% CI: 1.03–4.80; *p*-value = 0.042) than those with exclusive breastfeeding.

### Nutritional status and clinical profile of RVA-positive undernourished children

As shown in Table 4, most of the RVA-positive children were suffering from severe wasting (33.3% - 14/42), mild-to-moderate underweight (26.4% - 187/708) and severe stunting (32.0% - 16/50). More than 50% of children with RVA presented one to five episodes of diarrhoea and vomiting was the primary symptom (30.7% - 153/499; *p*-value = 0.007) (Table 4). In the sample, RVA infection was more common in HIV-positive children than HIV-negative (30.9% - 17/55 versus 25.5% - 132/518), but without significative differences (*p*-value > 0.05) (Table 4). About 22% of the positive children were hospitalized between 1 and 4 days (22.1% - 64/289; *p*-value = 0.017) (Table 4).

**Table 4:** Clinical characteristics and frequencies of undernourished children infected by RVA

Clinical characteristic	***N*** = 842	Positive RV (***n*** = 229)	% (Pos)	***P***-value
**Types of undernutrition**				
**Wasting**				–
Mild-to-Moderate	110	31	28.2	
Severe	42	14	33.3	
**Underweight**				–
Mild-to-moderate	708	187	26.4	
Severe	0	0	0.0	
**Stunting**				
Mild-to-Moderate	108	23	21.3	
Severe	50	16	32.0	
**Diarrhoea episodes in 24 h**				0.224
1–3	238	63	26.5	
4–5	332	99	29.8	
≥ 6	196	45	23.0	
Unknown/ missing	76	22		
**Vomit**				**0.007**
Yes	499	153	30.7	
No	337	75	22.3	
Unknown/ missing	6	1		
**HIV status**				0.383
Positive	55	17	30.9	
Negative	518	132	25.5	
Unknown/ indeterminate	269	80		
**Duration of Hospitalization (days)**				**0.017**
1–4	289	64	22.1	
5	77	15	19.5	
≥ 6	160	18	11.3	
Unknown/ missing	316	132		

-: Not applicable

### Sub-analysis of children that presented with triple disease burden (HIV, undernutrition and RVA)

The sub-analysis of the 7.4% (17/299) of the children that have presented triple burden of disease, showed that most of them were less than 12 months (76.5%); 82.4% were from Maputo and 11.8% from Nampula provinces, 76.5% were living in crowded households (with 5 or more members). Regarding clinical variables, 43.8% had one to three diarrhoeal episodes, 63.2% were underweighted, 31.6% wasted and 60% hospitalized between 1 and 4 days (Table 5).

**Table 5:** Characteristic and frequency of children with undernutrition, HIV and RVA

Clinical characteristic	Categories	***n*** = 17	HIV and RVA positive (%)
**Age in months**			
	0–11	13	76.5
	12–23	4	23.5
	24–59	0	0.0
**Province**			
	Maputo	14	82.4
	Nampula	2	11.8
	Zambézia	1	5.9
	Sofala	0	0.0
**Number of family members**			
	<5	4	23.5
	≥5	13	76.5
**Diarrhoea episodes in 24 h**			
	1–3	7	43.8
	4–5	6	37.5
	≥6	3	18.8
	Unknown/ missing	1	
**Vomit**			
	Yes	4	23.5
	No	13	76.5
**Undernutrition**^**a**^			
	Underweight	12	63.2
	Wasting	6	31.6
	Stunting	3	15.8
**Hospitalization (in days)**			
	1–4	3	60.0
	5	1	20.0
	≥6	1	20.0
	Unknown/ missing	12	

^a^ The sample size for undernutrition variable is 21 due to children that have more than one type of undernutrition

## Discussion

In the present analysis, we investigated the frequency and risk factors related to RVA infection in undernourished children under 5 years old with diarrhoea enrolled under the National Diarrhoea Surveillance in Mozambique. Our findings show that over one-quarter (27.2%) of children were infected with RVA. However, the frequency was twice higher before widespread implementation of the RVA vaccine (42.7% versus 12.2%). It appears that the introduction of the vaccine is effective to prevent RVA infection among undernourished children as the frequency decreased in the post-vaccine period (12.2%).

A study conducted in the southern region of Mozambique before the RVA vaccine introduction, found a lower frequency of RVA infection (37.7%) in underweighted children from rural and urban areas, compared with our results (80.7% data not shown) [19]. This difference can be explained by divergent methodological approaches, while de Deus et al. only included children with moderate-to-severe underweight, the present analysis assessed mild-to-severe underweight [19]. Besides that, de Deus et al. only assessed children from southern Mozambique, while in this analysis we included children from the northern and central provinces (Nampula and Zambézia), where the prevalence of undernutrition is known to be higher [12].

A study conducted in Angola reported a lower prevalence of RVA infection (13.08–31.06%) in undernourished children before the introduction of the RVA vaccine compared with the present analysis (42.7%). This finding may be explained by the higher proportion of undernourished children in Mozambique (43%) compared with Angola (38%) [7, 12, 29].

Another study conducted in Zambia aiming to determine RVA infection in hospitalized children reported a higher rate of RVA infection in well-nourished children compared with undernourished (27.6% versus 19.3%) respectively [10]. The proportion of undernourished children with RVA positive was also lower than in the present analysis.

In Mozambique, the Rotarix^®^ vaccine (GlaxoSmithKline Biologics, Belgium) was introduced in the National Expanded Program on Immunization in September 2015. In 2017, de Deus et al. determined the early impact of RVA vaccine through ViNaDia using the general sample without considering nutritional status. Comparing results by de Deus et al to our analysis, the previous one reported a lower RVA infection rate in 2015 (40.2% versus 42.7%), but not in 2016 (13.2% versus 12.2%) nor in 2017 (13.5% versus 9.1%) [20]. However, it is important to highlight that de Deus et al analysis was done until June 2017. In addition, it seems that undernourished children contributed significantly to the high rate of RVA infection observed by de Deus et al analysis as they represented more than 60% of the total population.

Our study suggests that children aged between 0 and 11 months are more likely to be infected by RVA when compared to children between 24 and 59 months of age. This finding probably shows that the infection in undernourished children commonly occurs during infancy and early childhood, as reported in Zambia and other low-in-come countries [10, 30]. This highlights that vaccination opportunity against RVA can not be missed in infants to avoid morbidity and mortality in this age group, since the double burden of diseases may happen before their first anniversary. Future studies are needed to understand the long-term impact of these double burden conditions on children’s lives.

Although this study did not show statistically significant differences between gender and RVA infection, several studies conducted in different countries have reported a higher proportion of RVA infection in males than in females [7, 18, 19, 31].

Interestingly, the risk of RVA infection in undernourished children was higher in Maputo the capital of the country, where most families have a higher wealth quintile compared to other provinces [12]. In addition, most of the undernourished RVA positive children families lived in brick houses. However, our surveillance has limited information about the conditions of water, sanitation and hygiene (WASH) in brick houses, leaving the question open, to what extent whether living in a brick house means having all the conditions to adequate access to WASH. According to a survey on a family budget (2015), Maputo has a higher number of family members (5.2) than Nampula (4.8) and Zambézia (4.7), which is in accordance with our analysis, which shows that overcrowded environments may provide a higher risk of RVA infection [32]. Also, most of the heads of households living in Maputo have a formal occupation, in contrast with those living in Nampula and Zambézia [32]. Based on this, our speculation is that most of the children from Maputo spend the day in kindergarten/home group care which increases the risk of diarrhoea and RVA infection when compared to children who spend the day at home [32, 33].

A cross-sectional study that included 71 households found a relationship between a high number of family members in household and risk for diarrhoea diseases [34]. In this study children’ living in households with more than four family members are at higher risk for diarrhoea RVA infection. A multi-country birth cohort study in countries with a high burden of diarrhoea and malnutrition (South Africa, Tanzania, Brazil, Peru, India, Nepal, Pakistan and Bangladesh) showed that children living in crowded households were more likely to have RVA diarrhoea [35]. Crowded households aligned with poor hygiene and sanitation can contribute significantly to the spread of enteric pathogens [36, 37].

Breast milk provides essential elements such as human oligosaccharides, secretory IgA, T and B lymphocytes to protect infants against enteric pathogens [38]. In this study, we found a high proportion of children infected with rotavirus fed exclusively breast milk or a combination of breast milk and formula. In Mozambique, national guidelines on breastfeeding practices comply with the WHO guidelines, which recommends exclusive breastfeeding until 6 months of age and continued breastfeeding until the child’s second anniversary with appropriate complementary solid food [12]. However, according to the last Demographic and Health Survey, only 43% of the Mozambican children were exclusively breastfed, with a median age of 1.3 months and for the complementary breastfeeding median age of 4.6 months [12]. This evidence shows that in Mozambique, younger children are exposed to complementary feeding earlier than the recommended. Our data confirmed that 45.5% (35/77) of children under 6 months of age received exclusive breastfeeding, while 36.5% (239/654) of children between 6 and 24 months of age were fed by breast milk and formula.

Early weaning and introduction of complementary food, has significant implication in the children’s health as their immune system is immature and may affect the intestinal function, increasing the risk of diarrhoea disease and stunting [39, 40].

Our binary logistic model seemed to show that different types of food are associated with RVA infection. However, the multiple logistic models, adjusted for age, suggests only a borderline significance when we compare other types of food with breast milk. This finding should be interpreted carefully. It is expected that children less than 6 months are more likely to have exclusive breastfeeding while others with more than 6 months are more likely to have a combination of breast milk and formula or other types of food. Thus, the association between the type of food and RVA infection tends to disappear when we introduced the age in the model. Some studies pointed out that the protective effects of breastfeeding seem to decrease with age [41–43]. In the literature, we found different variables to express the definitions of breastfeeding, age of children, and study designs, making comparisons and interpretations of the findings of different studies very difficult [43].

Most of the positive children had four to five episodes of diarrhoea in less than 24 h. Vomiting has been reported as one of the main symptoms of RVA infection, which is consistent with our results in undernourished children [19, 44, 45]. However, it is important to highlight that these studies were conducted without considering the children’ nutritional status.

The fact that 7.4% of the infected children were HIV positive indicates a triple burden to address (undernutrition, HIV and RVA infection). According to the Malaria, HIV/AIDS, and Immunization Indicator Survey in Mozambique women in reproductive age present the higher prevalence of HIV infection [46]. Which has a significant impact on increasing the rate of HIV infection in children due to vertical transmission.

While HIV contributes to the reduction of CD4+ T cells and the role of the immune system, which requires greater energy needs, undernutrition leads to immune dysfunction increases the risk of infections, poor response to vaccination and low antiretroviral treatment efficacy leading to high mortality [47]. RVA infection associated with this double burden diseases will generate synergism increasing the chances of developing Acquired Immunodeficiency Syndrome (AIDS) and chronic undernutrition, which can lead to the children’ mortality [48].

The majority of children who presented undernutrition, HIV and RVA infection were infants (< 12 months) from Maputo and Nampula provinces. Maputo was reported as having a high prevalence of HIV infection (16.9%) while Nampula province was reported as having a high prevalence of chronic undernutrition (55.3%) [12, 46]. Our data show that children living in both provinces are exposed to undernutrition and co-infections during the first year of life. Future studies are needed to understand this relationship and the long-term consequences of these conditions for the children.

Hospitalization of undernourished children is frequently associated with longer stay in healthcare facilities and a higher risk of death, mainly if associated with gastroenteritis [49]. However, in this study, most of the children were hospitalized for 1 to 4 days.

The findings of this study shows a high proportion of children undernourished with diarrhoea infected by RVA even after the vaccine introduction. This finding suggests that there is a need to evaluate if the treatment of the children affected by the above-mentioned conditions follows the recommended guidelines in order to understand the impact on the morbidity and mortality associated in children.

The major limitation of this study is the high number of missing data on the variables, even with the continuous training staff at the sentinel site to improve data quality collection. Additionally, the design of the study included only children who looked for health services care (inpatient and outpatient) and consequently more likely to have a severe infection and be undernourished. This is a cross-sectional study, which does not allow measuring the causality between variables and the RVA infection. Future studies should consider the possibility to include a community approach to understand better the real situation of RVA infection and the role of undernutrition in case-control or longitudinal studies.

## Conclusion

The present study showed that the frequency of RVA infection in undernourished children was high in the pre-vaccine period but declined following the RVA vaccine introduction in Mozambique. RVA infection risk factors were age (0–11 months), province (Maputo city), and crowed households (with five or more members). In addition, a high proportion of RVA infection was observed in children with severe wasting. A triple burden of disease: undernutrition, RVA and HIV were observed and highlights the need to conduct follow-up studies to understand the long-term impact of these conditions in children’s development.

## Data Availability

The raw data are available upon reasonable request from the corresponding author.

## Abbreviations

AIDS: Acquired Immunodeficiency Syndrome
AOR: Adjusted Odds Ratio
CI: Confidence interval
DFG: Deutsche Forschungsgemeinschaft
EFINTD: European Foundation Initiate into African Research in Neglected Tropical Diseases
ELISA: Enzyme Linked ImmunonoSorbent Assay
FNI: *Fundo Nacional de Investigação*
GEMS: Global Enteric Multicenter Study
HCB: *Hospital Central da Beira*
HCM: *Hospital Central de Maputo*
HGJM: *Hospital Geral José Macamo*
HGM: *Hospital Geral de Mavalane*
RVA: group A rotavirus
HIV: Human Immunodeficiency Virus
HGQ: *Hospital Geral de Quelimane*
HCN: *Hospital Central de Nampula*
HAZ: Height-for-age Z-scores
INS: *Instituto Nacional de Saúde*
NA: Not applicable
OR: Odds Ratio
SPSS: Statistical Package for Social Sciences
ViNaDia: National Diarrhoea Surveillance
WASH: Water Sanitation and Hygiene
WAZ: Weight-for-age Z-scores
WHZ: weight-for-height Z-scores
WHO: Word Health Organization
